# Age and dose dependent changes to the bone and bone marrow microenvironment after cytotoxic conditioning with busulfan

**DOI:** 10.3389/fcell.2024.1441381

**Published:** 2024-07-30

**Authors:** Nastaran Abbasizadeh, Christian S. Burns, Ruth Verrinder, Farhad Ghazali, Negar Seyedhassantehrani, Joel A. Spencer

**Affiliations:** ^1^ Department of Bioengineering, University of California, Merced, Merced, CA, United States; ^2^ Center for Cellular and Biomolecular Machines, University of California, Merced, Merced, CA, United States; ^3^ Health Sciences Research Institute, University of California, Merced, Merced, CA, United States

**Keywords:** hematopoietic cell transplantation, bone marrow microenvironment, hematopoietic recovery, bone remodeling, cytotoxic conditioning intensity, hematopoietic aging

## Abstract

Preparative regimens before Hematopoietic Cell Transplantation (HCT) damage the bone marrow (BM) microenvironment, potentially leading to secondary morbidity and even mortality. The precise effects of cytotoxic preconditioning on bone and BM remodeling, regeneration, and subsequent hematopoietic recovery over time remain unclear. Moreover, the influence of recipient age and cytotoxic dose have not been fully described. In this study, we longitudinally investigated bone and BM remodeling after busulfan treatment with low intensity (LI) and high intensity (HI) regimens as a function of animal age. As expected, higher donor chimerism was observed in young mice in both LI and HI regimens compared to adult mice. Noticeably in adult mice, significant engraftment was only observed in the HI group. The integrity of the blood-bone marrow barrier in calvarial BM blood vessels was lost after busulfan treatment in the young mice and remained altered even 6 weeks after HCT. In adult mice, the severity of vascular leakage appeared to be dose-dependent, being more pronounced in HI compared to LI recipients. Interestingly, no noticeable change in blood flow velocity was observed following busulfan treatment. *Ex vivo* imaging of the long bones revealed a reduction in the frequency and an increase in the diameter and density of the blood vessels shortly after treatment, a phenomenon that largely recovered in young mice but persisted in older mice after 6 weeks. Furthermore, analysis of bone remodeling indicated a significant alteration in bone turnover at 6 weeks compared to earlier timepoints in both young and adult mice. Overall, our results reveal new aspects of bone and BM remodeling, as well as hematopoietic recovery, which is dependent on the cytotoxic dose and recipient age.

## Introduction

Hematopoietic Cell Transplantation (HCT) is a common therapeutic approach for hematologic malignancies such as leukemia, multiple myeloma, lymphoma and non-malignant diseases (Kanate et al., 2020; D Souza et al., 2017; Gyurkocza and Sandmaier, 2014; Chhabra et al., 2018; Du and Cao, 2018). Successful donor cell engraftment after HCT necessitates cytotoxic preconditioning (Gyurkocza and Sandmaier, 2014; Nagler et al., 2019). Historically, Myeloablative Conditioning (MAC) has been considered a standard preconditioning regimen for patients in need of HCT(7). The inherent toxicity and non-relapse mortality associated with MAC, however, limits its use to a select group of patients (Chiesa and Veys, 2012; Yanada et al., 2022). To address these limitations, a more tolerable preparative regimen, known as Reduced Intensity Conditioning (RIC), was developed for less fit patients (Chiesa and Veys, 2012; Sengsayadeth et al., 2015; Çiftçiler et al., 2019). Clinical data indicate that RIC has a lower cumulative incidence of chronic graft-versus-host disease but similar overall survival compared to MAC, positioning it as a potential alternative treatment (Chhabra et al., 2018). Insufficient conditioning, however, can lead to early disease relapse after transplantation due to the lack of graft vs malignancy effect from mixed chimerism (Cavazzana-Calvo et al., 2007; Barrett and Battiwalla, 2010). Therefore, it is important to fine tune the intensity of RIC to provide the most effective clinical outcomes after HCT.

Cytotoxic preconditioning is known to disrupt the hematopoietic and non-hematopoietic compartments of the bone and bone marrow (BM) microenvironments regardless of the preparative regimen used (Slayton et al., 2007; Zou et al., 2016; Chen et al., 2019; Kouam et al., 2019). After treatment, BM vasculature can undergo significant changes such as the dilation and fusion of sinusoidal vessels and a temporary decrease in vessel frequency (Li et al., 2008; Zhou et al., 2017; Fletcher et al., 2023). Disruption in both osteoblast and osteoclast activities result in increased rates of bone resorption (Kondo et al., 2009; Zou et al., 2016). Furthermore, mesenchymal stem cells after exposure to low irradiation have been found to exhibit a shift in their differentiation capacity towards less adipocytes and more osteogenic cells (Preciado et al., 2018).

In addition to conditioning regimens, the aging process is also known to disrupt the functionality of the BM niche. Age-related changes such as a notable decrease in osteoprogenitors, a decline in the number of the metaphyseal blood vessels, and alterations in the differentiation and proliferation of mesenchymal stromal cells, negatively impact hematopoiesis (Ogawa et al., 2000; Kusumbe et al., 2014; Porto et al., 2015; Kusumbe et al., 2016; Lee et al., 2019). Furthermore, elevated levels of intracellular reactive oxygen species (ROS), accumulation of the DNA damage, upregulation of pro-inflammatory cytokines such as IL-6, NF-κB, C-reactive protein or dysregulated DNA methylation patterns at the genes contributing to the lymphoid and myeloid balancing are the other alterations that Hematopoietic Stem Cells (HSCs) experience during aging (Cohen et al., 1997; Hasegawa et al., 2000; Rübe et al., 2011; Beerman et al., 2014; Kovtonyuk et al., 2016; Connor et al., 2018). Consequently, the host response to the preparative regimen is contingent on both the intensity of cytotoxic treatment and age of the recipient.

In this study, we used two doses of 1,4-Butanediol dimethanesulfonate (busulfan) called Low Intensity (LI) and High Intensity (HI) to condition the BM niche in both young and adult mice before HCT. Busulfan, a DNA alkylating drug, is commonly used in combination with cyclophosphamide to treat leukemia (Socié et al., 2001; Ciurea and Andersson, 2009; Kebriaei et al., 2018). Various doses of busulfan have been previously used in myeloablative or non-myeloablative preconditioning (Ashizuka et al., 2006; Montecino-Rodriguez and Dorshkind, 2020; Garcia-Perez et al., 2021). Our research reveals significant alterations to the BM microenvironment after busulfan treatment that are dependent on both age and dose. This data provides unique insight into BM recovery, offering valuable information that could contribute to the development of more tailored preparative treatments for HCT patients within specific age groups.

## Materials and methods

### Animals

Male C57BL/6J, C57BL/6 CD45.1 (B6.SJL-Ptprca Pepcb/BoyJ) and C57BL/6-Tg(UBC-GFP)30Scha/J transgenic mice were purchased from Jackson laboratory. Mice were bred and housed in the Department of Animal Research Services (DARS) at UC Merced. The animal study received approval from the Institutional Animal Care and Use Committee (IACUC) at UC Merced.

### Busulfan treatment and whole BM cell transplantation

We used 4–6 weeks (young) and 16–20 weeks (adult) old male C57BL/6 CD45.1 and C57BL/6J mice as recipients. To investigate the effect of variable RIC dosage, mice received either 40 mg/kg (LI) or 80 mg/kg (HI) dose of Busulfan (Cayman Chemicals Company; 14,843) *via* intraperitoneal injection (IP). Busulfan solution was prepared immediately before injection as previously described (Montecino-Rodriguez and Dorshkind, 2020). Briefly, busulfan crystals were dissolved in DMSO (Sigma Aldrich; 472,301) and Ca+/Mg + free PBS (gibco; 2,003,901) was added to the solution to make a final drug concentration of 1 mg/mL in 10% DMSO. The working solution was filtered through a 0.22 µm syringe filter (Fisherbrand; R7PA99681) and was administered to mice in separate doses of 20 mg/kg per day on two (LI) and four (HI) consecutive days.

One day following busulfan conditioning, 8–12 weeks UBC-GFP transgenic donor mice were euthanized by CO_2_ inhalation and cervical dislocation. Long bones were collected, cleaned, and crushed in Flow Cytometry Staining (FACS) buffer. The cell mixture was filtered through a 40 μm filter into a 50 mL falcon tube and spun at 1,500 rpm for 5 min at 4°C. The supernatant was aspirated, and the pellet was resuspended in Ammonium-Chloride-Potassium (ACK) lysis buffer to remove erythrocytes. The reaction was stopped after 1 min incubation by adding FACS buffer and cells were washed by centrifuging at 2000 rpm for 3 min at 4°C. Cells were resuspended in PBS and a cell count was performed using a hemocytometer and Trypan Blue (gibco, 15250-061) staining. Finally, a suspension of 1 × 10^6^ cells/mL was administered by retroorbital injection.

### Flow cytometry chimerism: peripheral blood and bone marrow

To collect peripheral blood, mice were kept under a heat lamp for a few minutes to increase blood circulation. The mice were transferred to a restrainer and a small incision was made over the ventral tail vein using a scalpel blade. Blood was collected (no more than 10 drops) and stored in blood collection tubes spray coated with K2EDTA (BD Microtainer; 365,974). Heparinized blood was added to 9 mL diH_2_O for RBC lysis and immediately after resuspension, 1 mL ×10 PBS was added to the solution to prevent white blood cells lysis. Cells were spun at 2000 rpm for 5 min at 4°C. The supernatant was removed, and the pellet was resuspended in 9 mL diH_2_O and 1 mL ×10 PBS to remove the remaining RBC. Cells were spun at 2000 rpm for 3 min at 4°C and 100 µL of the sample was aliquoted into 96V-bottom wells for FACS staining. Cells were washed with 100 µL of FACS buffer and centrifuged at 2000 rpm for 3 min at 4°C. The supernatant was removed, and cells were stained with 50 µL of staining cocktail containing APC/Cy7 CD45.2 (1:100, Biolegend; 109,824) and PerCP/Cy5 CD45.1 (1:100, Biolegend; 110,728) in the dark on ice. After 15 min incubation, cells were washed with FACS buffer, spun, and resuspended in 200 µL FACS buffer for flow cytometry chimerism.

To collect the whole BM cells, mice were euthanized on day 42 post-HCT and the same procedure as the whole BM transplantation described previously was performed to collect cells. Cells were then resuspended in PBS and a suspension of 1 × 10^6^ cells/mL was collected for staining. After 15 min incubation with APC/Cy7 CD45.2 (1:100) and PerCP/Cy5 CD45.1 (1:100) cells were washed with FACS buffer, spun and resuspended in 200 µL FACS for flow cytometry chimerism (Chicana et al., 2022). During flow cytometry, to evaluate chimerism, CD45.2 donor cells were gated against CD45.1 recipient cells, followed by gating GFP *versus* CD45.2 to validate GFP expression in UBC-GFP mice.

### Two-photon *in vivo* and *ex vivo* imaging

*In vivo* calvaria and *ex vivo* long bone imaging was performed with a custom-built two-photon intravital microscope (BLIQ Photonics). A ×25 fluid immersion objective (Olympus; XLPLN25XWMP2, NA = 1.05) was used for all images, with an approximate field of view (FOV) of 317 µm by 159 µm. During live imaging, two tunable femtosecond lasers, MaiTai (Spectra Physics; MaiTai eHP DS) and Insight (Spectra Physics; Insight X3), were tuned to 800 nm and 950 nm to observe 70 kDa Rhodamine-B Dextran (ThermoFisher, D1841) and GFP + cells signals, respectively. For *ex vivo* imaging, the MaiTai and Insight lasers were initially tuned to 950/1,220 nm to excite GFP, Alexafluor 647 conjugated vascular antibodies, and Second Harmonic Generation (SHG), respectively. The long bones were imaged a second time at 800 nm to excite Calcein and Alizarin. Videos were recorded at 30 frames per second and images were generated by averaging 30 frames.

For *in vivo* visualization of the calvaria BM, anesthesia was induced *via* initial inhalation of 3%–4% isoflurane with 100% O_2_ at 1 L/min that was reduced to 1.5% as maintenance. The skull was secured in a custom head mount that was equipped with a heating pad to maintain the animal body temperature during the procedure. After shaving top of the head, a small incision was made along the sagittal and lambda suture of the skull to expose the calvarium as described before (Sipkins et al., 2005).

For *ex vivo* imaging of the long bone, mice were injected retro-orbitally with Alexafluor 647 conjugated vascular antibodies (CD31; 102,516, Biolegend, Sca-1; 108,118, Biolegend, VE-Cadherin; 138,006, Biolegend) 30 min before intracardiac perfusion. To study bone remodeling, Calcein (30 mg/kg, Sigma; SLCF7304) and Alizarin (20 mg/kg, Sigma; SHBL6801) were administered 48 h and 30 min before imaging to track bone turnover based on the ratio of dye1 (Calcein; marks the old bone front) to dye2 (Alizarin; marks the new bone front). Note that in some animals, leptin receptor antibody was also administered *via* injection 1 hour prior to perfusion. However, due to insufficient signal strength, it was excluded from the protocol. Mice were perfused with 1x PBS to wash out the blood followed by 4% paraformaldehyde (PFA, Fischer Chemical; 1,638,384) to internally fix the tissue. Subsequently, femurs were harvested and fixed in 4% PFA for 30 min, at 4°C. The bones were then washed with 1X PBS, immersed in 30% sucrose (Sigma; SLCC8492) for 1 h, frozen in optimal cutting temperature (OCT, Fisher Scientific; 4,585) compound and kept at −80°C. Samples were shaved using a cryostat (LEICA CM 1860) equipped with a high-profile blade (Leica; 3,802,121) to expose the BM region.

### Image quantification

We used Fiji (ImageJ 1.53t) for image processing including quantification of vessel permeability, leakage, morphological changes to the blood vessel, evaluating bone turnover rate, and quantifying GFP + donor cells engraftment in the recipient BM. Custom scripts in MATLAB (2020a) were used to calculate BM blood flow velocity (Wu et al., 2021). In order to study changes to the vascular system in the calvaria BM, 70 kDa Rhodamine-B Dextran was injected retro-orbitally during *in vivo* imaging. The blood vessel permeability was measured during the first 30 s post dye injection and was quantified based on the change in fluorescence intensity outside of blood vessels as a function of time as described before (Itkin et al., 2016; Ishii, 2018; Chicana et al., 2022). Vascular leakage was measured through z stack images (2 µm z step) taken 10 min after dye administration as described before and was calculated based on the ratio between the fluorescent intensity in the perivascular space to the fluorescent intensity in the adjacent vascular lumen (Chicana et al., 2022). Note that vessel permeability reflects the rate at which small molecules exit blood vessels and fill the surrounding perivascular space, whereas leakage is the ratio of fluorescent dye in the perivascular space and vascular lumen after reaching equilibrium. Blood flow velocity was calculated by recording 30 s videos of blood flow in the BM calvaria and then utilizing Line Scanning Particle Image Velocimetry (LSPIV) implemented in a custom MATLAB script to calculate blood flow velocity as previously described (Kim et al., 2012; Wu et al., 2021).

To measure vascular density, blood vessel images were color thresholded in Fiji (ImageJ 1.53t). The resulting binary image was despeckled, the binary fill hole function was applied, and a Python (3.7.6) script was used to calculate the ratio of the total blood vessel volume to total BM volume. We defined vessel density as the total space of the BM occupied by blood vessels. To study bone remodeling, the double-staining approach was performed by using Calcein (dye1) and Alizarin (dye2). The ratio of dye1/dye2 was calculated by measuring the total Calcein pixel area to the total Alizarin pixel area in each FOV. Based on the dye ratio, fractions of the cavity type (D-type; >0.75, M-type; 0.25–0.75, R-type; <0.25) were quantified as described previously (Christodoulou et al., 2020). To evaluate the early homing of donor cells, max intensity projections (MIP) of the long bone were taken and the number of GFP + cells in the BM on day 2 post-HCT was manually counted. Representative samples of BM leakage and long bone images were generated by taking MIP of BM regions and contrast/enhancement adjustment was applied for display purposes.

### Statistical analysis

In our study, a G*Power statistical power analysis (α = 0.05, power = 0.95) determined that a minimum of three mice per group is required for our analysis. Mice that experienced unsuccessful injections or showed signs of distress after retroorbital injection of Rhodamine-B Dextran during *in vivo* measurements were excluded from the experiments to ensure that only data from stable and properly injected mice were included in our analysis. Graphs and statistical analyses were generated using GraphPad Prism 9.0. Ordinary one-way ANOVA to test differences between study groups. A normality test was performed to assess the normal distribution of the data. A *p*-value less than 0.05 was considered to be statistically significant (*p < 0.05, **p < 0.01, ***p < 0.001; ****p < 0.0001).

## Results

### Dose and age dependent hematopoietic recovery following busulfan conditioning and HCT

**FIGURE 1:**
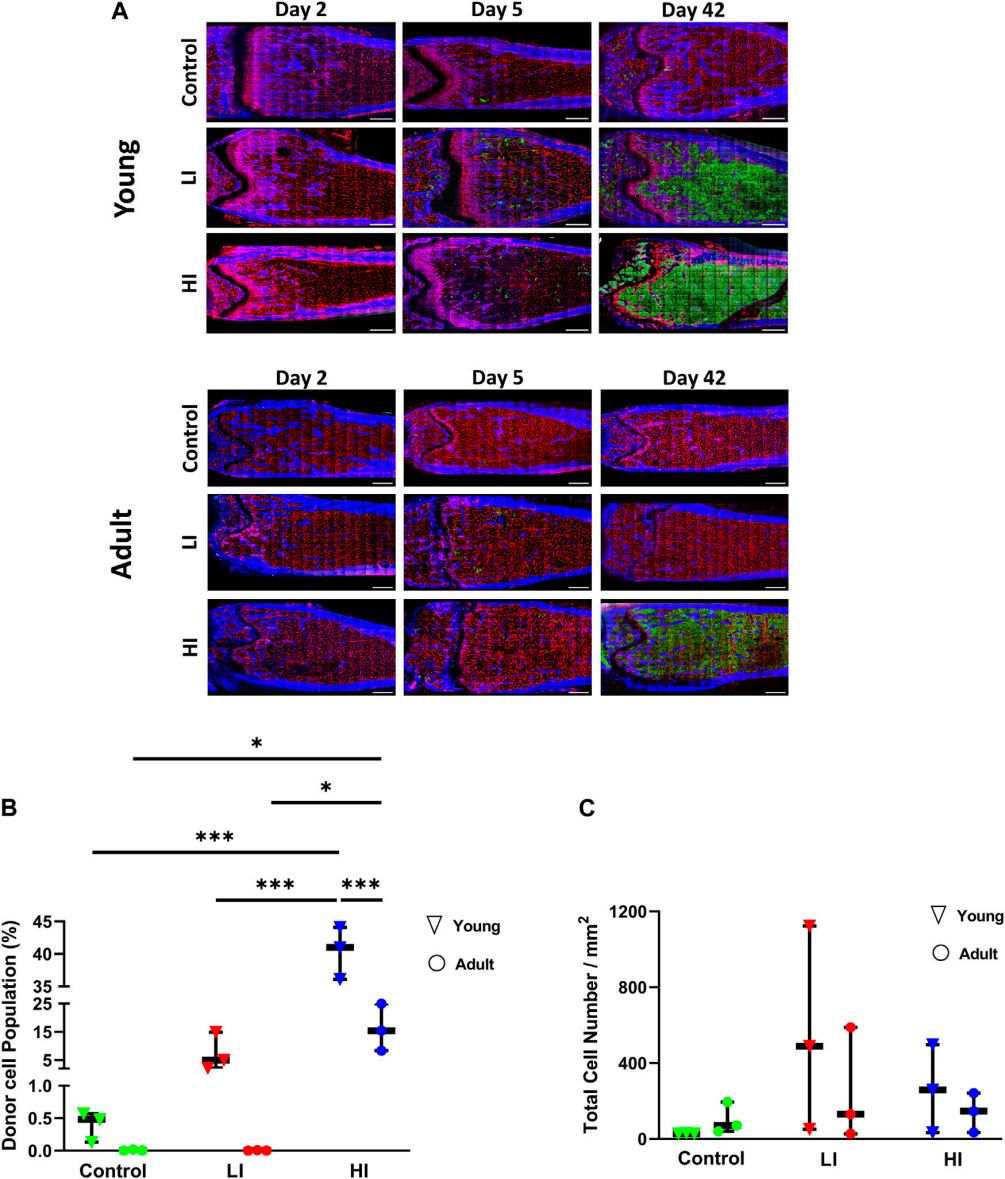
LI and HI Regimens Induce Dose and Age Dependent Hematopoietic Recovery in Young and Adult Mice. **(A)** Representative images of the transplanted BM in controls and LI/HI conditioned young (top) and adult (bottom) mice over time; Red: blood vessel (Alexafluor 647 conjugated CD31, Sca-1, VE-Cadherin), Blue: Bone (Second Harmonic Generation (SHG)), Green: GFP + cells; scale bar: 500 μm; **(B)** Quantification of donor GFP + cells in controls, and LI/HI conditioned BM 42 days post-HCT in young and adult mice; **(C)** Evaluation of donor cell homing in the long bone BM 2 days post-HCT in young and adult mice. Green: Control, Red: Low Intensity (LI), Blue: High intensity (HI). n = 3, *p < 0.05, ***p < 0.001.

To longitudinally investigate changes to the BM microenvironment as a function of chemotherapy dose and animal age, we created a HCT model incorporating busulfan preconditioning with low and high intensity in young (4–6 weeks old) and adult (16–20 weeks old) mice (Supplementary Figure S1A). After preconditioning, we transplanted whole BM cells from GFP + donor mice (Ubiquitin-GFP mice) and evaluated the bone and BM microenvironment as well as hematopoietic recovery on days 2, 5, and 42 post-HCT (Supplementary Figure S1A; Supplementary Videos S1, S2). Consistent with previous literature, BM images and flow cytometry of BM isolates on day 42 post-HCT revealed a higher accumulation of GFP + donor cells in busulfan conditioned young mice, particularly in the HI group (*p* < 0.001), compared to controls (Figures 1A,B; Supplementary Figure S1B) (Garcia-Perez et al., 2021). In the adult mice, only mice that received HI conditioning revealed effective hematopoietic engraftment 42 days after transplantation (*p* < 0.05; Figures 1A,B; Supplementary Figure S1C). Comparison of donor chimerism on day 42 in peripheral blood of young and adult mice was consistent with the BM imaging and chimerism analysis (Supplementary Figure S1D). In addition to dosage, hematopoietic recovery was dependent on the recipient’s age particularly in HI group (BM, *p* < 0.001; peripheral blood, *p* < 0.05; Figure 1B; Supplementary Figure S1D). Early homing of hematopoietic donor cells was evaluated by manual counting of GFP + cells in both the long bones and calvaria on day 2 post-HCT. No statistically significant difference was observed between young and adult mice suggesting that hematopoietic engraftment is more age dependent than early homing (Figure 1C; Supplementary Figure S1E).

### Dose and age dependent alteration in the vascular morphology and functionality following busulfan conditioning and HCT

**FIGURE 2:**
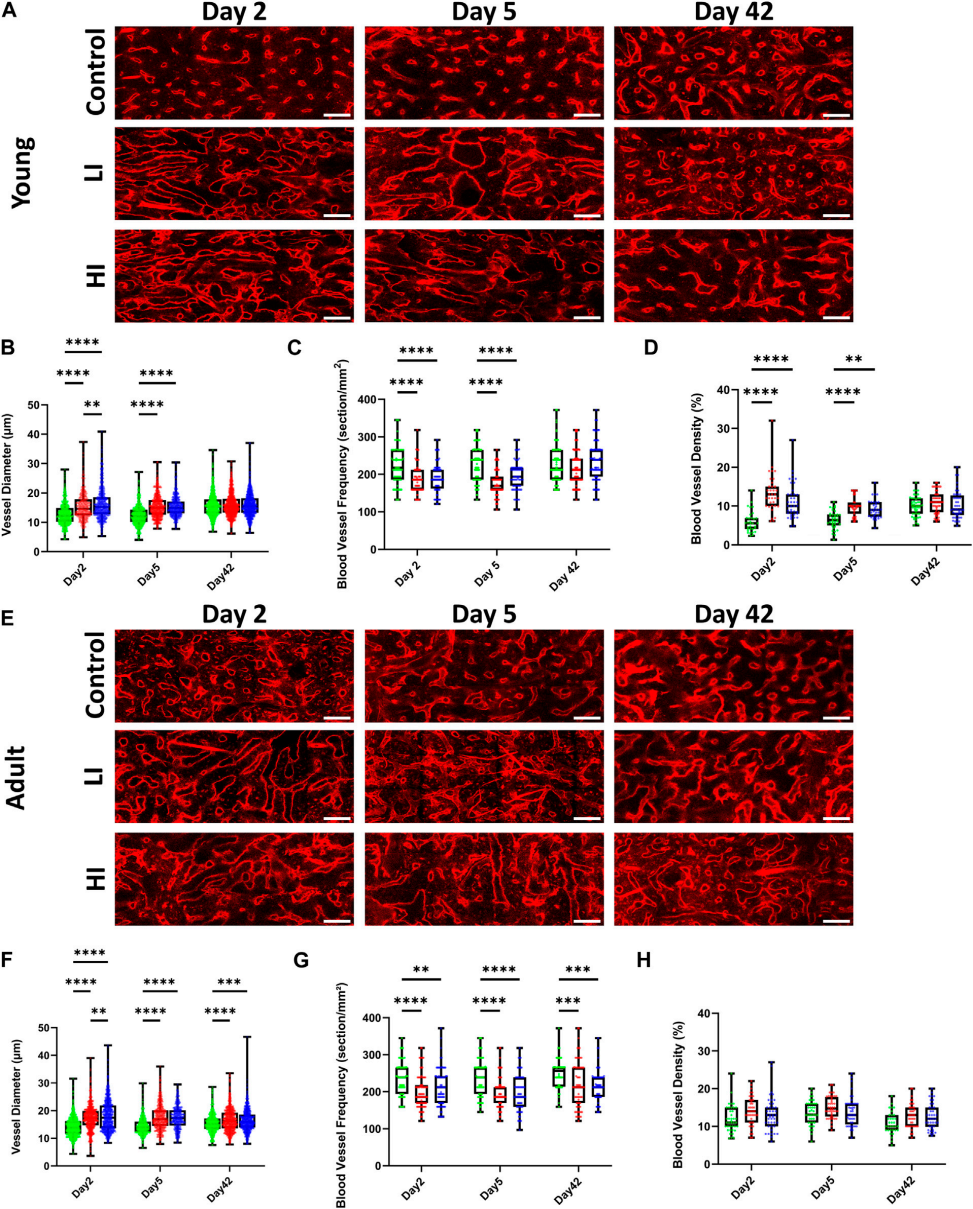
LI and HI Regimens Induce Morphological Alterations in BM Blood Vessels in Young and Adult Mice. **(A)** Representative images of the long bone BM blood vessel in controls and LI/HI conditioned young mice over time; Red: blood vessel (Alexafluor 647 conjugated CD31, Sca-1, VE-Cadherin); scale bar: 50 μm; **(B–D)** Quantification of the vascular diameter **(B)**, frequency **(C)**, and density **(D)** in controls and LI/HI conditioned young mice over time; **(E)** Representative images of the long bone BM blood vessel in controls and LI/HI conditioned adult mice over time; scale bar: 50 μm; **(F–H)** Quantification of the vascular diameter **(F)**, frequency **(G)**, and density **(H)** in controls and LI/HI conditioned adult mice over time. Green: Control, Red: Low Intensity (LI), Blue: High intensity (HI). n = 3, **p < 0.01, ***p < 0.001; ****p < 0.0001.

To evaluate the morphology of the BM vascular system after busulfan conditioning and HCT, we quantified the blood vessel diameter, frequency, and density. In young mice at early timepoints (days 2 and 5) post-HCT, blood vessel diameter was markedly increased while the vessel frequency was decreased compared to the controls (*p* < 0.0001), but they largely rescued by day 42 after transplantation (Figures 2A–C). Furthermore, at early days post-HCT, the vessel density was substantially increased in LI (*p* < 0.0001 on days 2 and 5) and HI (*p* < 0.0001 on day 2 and *p* < 0.01 on day 5) but was comparable to controls by day 42 (Figures 2A,D). Unlike young mice, vascular diameter (LI; *p* < 0.0001 and HI; *p* < 0.001; Figures 2E,F) and frequency (*p* < 0.001; Figures 2E,G) remained abnormal after 42 days in busulfan treated adult mice, suggesting an age-dependent delay in BM regeneration. Additionally, vessel density remained consistent across all study groups at all timepoints in adult mice (Figures 2E,H).

**FIGURE 3:**
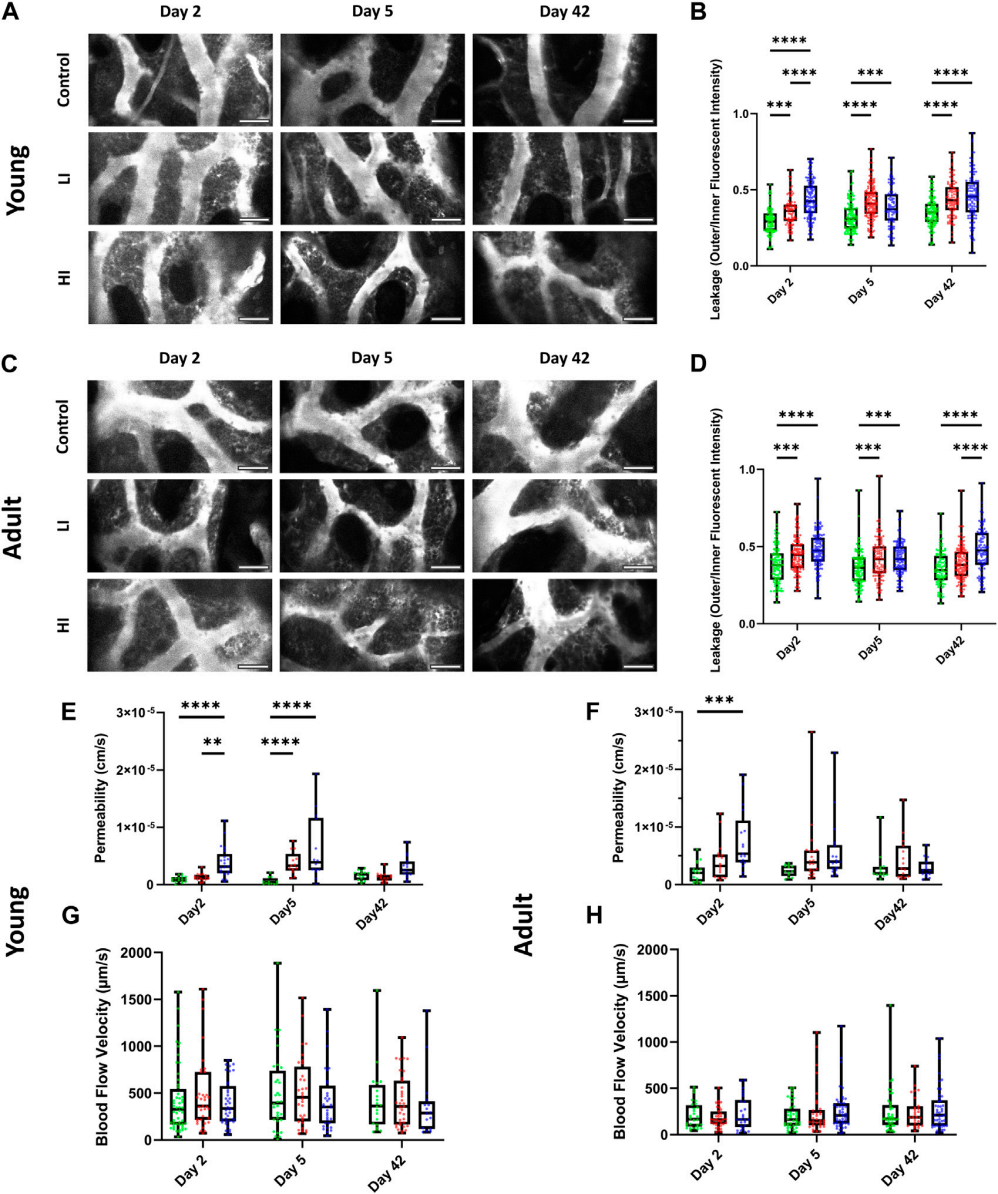
LI and HI Regimens Induce Disruption in BM Blood Vessel Barrier in Young and Adult Mice. **(A, B)** Representative images **(A)** and quantification of calvarial BM blood vessel leakage **(B)** in controls and LI/HI conditioned young mice; White: blood vessel (Rhodamine B Dextran, 70 kDa); scale bar: 50 μm; **(C, D)** Representative images **(C)** and quantification of calvarial BM blood vessel leakage **(D)** in controls and LI/HI conditioned adult mice; scale bar: 50 μm; **(E, F)** Quantification of calvarial BM blood vessel permeability in young **(E)** and adult **(F)** mice; **(G, H)** Quantification of calvarial BM blood flow in young **(G)** and adult **(H)** mice. Green: Control, Red: Low Intensity (LI), Blue: High intensity (HI). n = 3, **p < 0.01, ***p < 0.001; ****p < 0.0001.

Next, we evaluated the functionality of the BM vascular system *in vivo* using two-photon intravital microscopy by measuring the blood vessel leakage, permeability, and blood flow velocity in calvarial BM after intravenous injection of Rhodamine-B Dextran (70 kDa) as previously described (Wu et al., 2021). In young mice, vascular leakage increased and remained elevated at least until day 42 post-HCT in LI and HI conditioned groups (*p* < 0.0001) compared to the controls (Figures 3A,B; Supplementary Videos S3–S5). In adult mice, increased leakage was long-lasting only in the HI group compared to the LI and control groups suggesting a dose-dependent effect on recovery of the blood-bone marrow barrier (*p* < 0.0001; Figures 3C,D; Supplementary Videos S6–S8). The dose-dependent effect of busulfan on individual vessel permeability in young mice emerged at the earliest timepoint (day 2 post-HCT) where higher permeability was observed in HI groups compared to LI (*p* < 0.01) and control groups (*p* < 0.0001; Figure 3E). On day 5, however, both LI and HI groups exhibited equally elevated permeability compared to the controls (*p* < 0.0001; Figure 3E). In the adult mice, busulfan conditioning caused elevated permeability only in the HI group on day 2 post-HCT (*p* < 0.001; Figure 3F). Regardless of the animal age, permeability returned to baseline levels by day 42 (Figures 3E,F). Interestingly, blood flow velocity in busulfan conditioned groups remained consistent with controls irrespective of the busulfan dose or age of the recipient (Figures 3G,H).

### Dose and age dependent bone remodeling following busulfan conditioning and HCT

**FIGURE 4:**
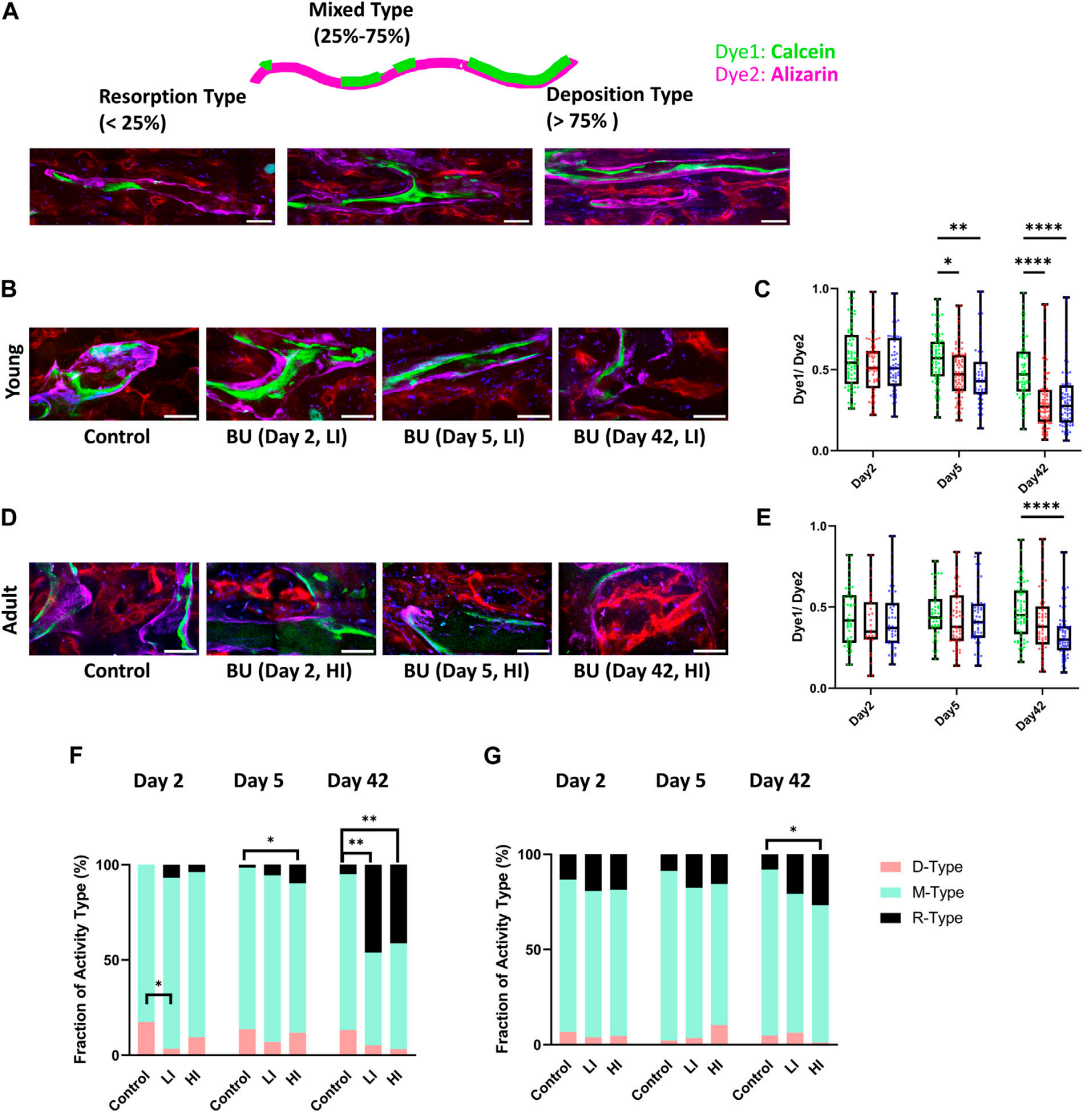
LI and HI Regimens Induce Bone Remodeling in Young and Adult Mice. **(A)** Schematic representation of the double calcium staining illustrating D-, M-, and R-type cavities; scale bar: 50 μm; **(B, C)** Representative images **(B)** and quantification **(C)** of dye1/dye2 ratio in controls and LI/HI conditioned young mice; **(D, E)** Representative images **(D)** and quantification of **(E)** dye1/dye2 ratio in controls and LI/HI conditioned adult mice; Images: Green: Calcein, Purple: Alizarin, Red: blood vessel (Alexafluor 647 conjugated CD31, Sca-1, VE-Cadherin); Graphs: Green: Control, Red: Low Intensity (LI), Blue: High intensity (HI). scale bar: 50 μm; **(F, G)** Quantification of fractions D-, M- and R-type cavities in young **(F)** and adult **(G)** long bone cavity. n = 3, *p < 0.05, **p < 0.01, ****p < 0.0001.

Recognizing that bone is a dynamic tissue that actively interacts with the hematopoietic system, we speculated that busulfan administration may induce bone remodeling. As described previously, we administered Calcein (dye1) and Alizarin (dye2), two calcium-binding dyes, 48 h and 30 min before imaging, respectively, to investigate the bone resorption-deposition profile based on the ratio of the two dyes (Figure 4A) (Christodoulou et al., 2020). In young mice, we observed a reduction in the dye1/dye2 ratio along the endosteum of the long bone cavity on day 5-post HCT in LI (*p* < 0.05) and HI (*p* < 0.01) groups which was more pronounced by day 42 in both groups (*p* < 0.0001) compared to the controls (Figures 4B,C). In adult mice, however, reduction in the dye1/dye2 ratio was not observed until day 42-post HCT and it only occurred in the HI group (*p* < 0.0001; Figures 4D,E). To further investigate the bone remodeling, we defined individual long bone cavities as Deposition type (D-type; dye1/dye2 > 75%), Mixed type (M-type; dye1/dye2 25%–75%), and Resorption type (R-type; dye1/dye2 < 25%) based on the ratio of the two dyes as described previously (Figure 4A; Christodoulou et al., 2020). Based on this classification, an increase in the R-type cavities was the determining factor for the decrease in the dye1/dye2 ratio after busulfan conditioning (Figures 4F,G). Additionally, a small but significant decrease in D-type cavities was observed with LI groups in young mice (Figure 4F).

## Discussion

In this project, we investigated the longitudinal impact of busulfan conditioning on the BM niche in the context of HCT, with a focus on how the intensity of conditioning regimens and animal age influence this dynamic process. It is known that the intensity of conditioning and the recipient age can affect the donor cell engraftment after HCT [(Garcia-Perez et al., 2021; Down et al., 1991; Van Os et al., 1993; Yanir et al., 2022)], but less is known about the direct impact these factors have on the BM microenvironment. In our experiments, a more intense chemotherapy dose improved long-term engraftment in both young and adult mice. This effect is more prominent in adult mice where high engraftment was observed in HI mice but very few cells survived in the BM of LI and controls on day 42. High intensity regimens are known to more effectively eradicate recipient hematopoietic cells compared to low intensity regimens, providing an open BM microenvironment for donor cell engraftment (Kenyon and Babic, 2018; Griffin et al., 2022). In addition, chemotherapy treatment leads to an efficient hematopoietic recovery through stimulating the secretion of GM-CSF, a crucial factor for the proliferation of donor cells (Sudo et al., 2021). We also observed an overall better engraftment in young groups compared to the corresponding adult groups as expected. Interestingly, long bone image analysis revealed similar early homing kinetics between groups regardless of chemotherapy dose or age, even though long-term engraftment was higher in young mice compared to adults. This discrepancy is associated with the age-dependent alterations in the BM that cause the BM to be less ideal for transplanted HSCs to engraft and repopulate, such as the downregulation of stromal cell-derived chemokines (e.g., SDF-1), reduction of arterioles and their HSC regulatory cells (e.g., arteriolar NG2+, and PDGFβ+ cells) in endosteal regions, and increased generation of ROS [(Kusumbe et al., 2014; Porto et al., 2015; Lee et al., 2019; Ellis et al., 2011; Maryanovich et al., 2018; Ho et al., 2019; Saçma et al., 2019; Singh et al., 2020)].

Given the critical role that the BM niche plays in supporting hematopoiesis, we subsequently examined busulfan-induced changes to different microenvironmental compartments of the BM. Consistent with other findings, we observed increased blood vessel leakage and permeability as well as changes in the blood vessel morphology, size, and number at early days after cytotoxic treatment (Kopp et al., 2005; Li et al., 2008; Hooper et al., 2009; Zhou et al., 2017; Kouam et al., 2019). In addition, our experiments unexpectedly show that despite significant hematopoietic reconstitution, the blood vessels remain leaky on day 42 post-HCT suggesting that full recovery of the blood-bone marrow barrier requires more than 6 weeks or may never fully recover. A persistent reduction in the number of endothelial cells observed 4 weeks after cisplatin chemotherapy supports our findings on the prolonged impact of chemotherapy on the blood vessel barrier (Lucas et al., 2013). It is worth noting that chemotherapy-driven endothelial damage can contribute to various clinical complications, such as sepsis, which is associated with high morbidity and mortality rates in patients (Karvunidis et al., 2012; Gudiol et al., 2021).

Furthermore, morphological changes to the blood vessel network rebounded in young mice by day 42 yet remained altered in adult mice, likely due to decreased or delayed regenerative potential of adult BM. A decline in the number and function of mesenchymal stem cells (MSCs), adipocyte accumulation, reduced angiogenesis, and increased secretion of inflammatory molecules are known age-associated factors that disrupt the BM microenvironment and contribute to delayed regeneration after cytotoxic damage (Ferrucci et al., 2005; Ferrucci et al., 2005; Naveiras et al., 2009; Siclari et al., 2013; Kusumbe et al., 2014; Mendelson and Frenette, 2014).

Unlike the early response of the vascular system to chemotherapy that begins as early as day 2, a shift in the balance of bone remodeling towards resorption appears delayed, particularly in adult mice, and becomes increasingly pronounced over time. In young mice, a general decrease in D-type bone (significantly in the LI group) and an increase in R-type bone were observed in the bone classification analysis, suggesting that the effects of chemotherapy may begin to manifest early after treatment. Alizarin Red and ALP staining of bone MSCs in young mice 3 days post-cyclophosphamide chemotherapy showed a decrease in osteoblastogenesis, supporting our observations (Zhao et al., 2017).

Similarly, localized irradiation has been found to reduce the ratio of osteoblasts area to bone surface area (OB/BS) while increasing the ratio of osteoclast area to bone surface area (OC/BS) after 2 weeks post-exposure and persists for a minimum of 12 weeks (Zou et al., 2016). Furthermore, the application of double bone labeling (Calcein/Alizarin) in femurs of mice subjected to Doxorubicin chemotherapy has revealed a decrease in the bone formation rate per bone surface (BFR/BS) and a reduction in the mineral apposition rate (MAR) (Yao et al., 2020). This dynamic change in bone turnover is attributed to increased osteoclast activity and decreased osteoblast activity following conditioning (Green et al., 2012; Yao et al., 2020).

Our results highlight that restoration of hematopoiesis through donor cell expansion does not necessarily require full recovery of the BM microenvironment and normal rates of bone turnover. While bone turnover is a complex and tightly regulated process that involves the coordinated activity of osteoclasts and osteoblasts, the restoration of hematopoiesis involves a variety of cells, signals, and resources that is independent of bone turnover in terms of timing and dynamics. In this regard, clinical data suggests that bone loss does not compromise hematopoietic recovery and patients undergoing HCT show successful BM repopulation despite significant bone density loss within 6 months post-treatment (Kang et al., 2000). While it is known that HSPCs require various components in the BM microenvironment to reconstitute hematopoiesis, additional research is needed to reveal the full extent of influence that each of these components exerts on hematopoietic recovery after transplantation (Lo et al., 2009; Lai et al., 2014).

Altogether, our findings highlight the distinct influence that chemotherapy dosage and recipient age have on the remodeling of the bone and BM niche and donor cell recovery after HCT. These insights contribute fundamental knowledge to clinical oncology, particularly in understanding dose-response relationships for cytotoxic therapy in cancer patients of various ages.

## Data Availability

The original contributions presented in the study are included in the article/Supplementary Material, further inquiries can be directed to the corresponding author.

## Supplementary material

SUPPLEMENTARY FIGURE S1 Dose and Age Dependent Donor Hematopoietic Recovery Following Busulfan Conditioning. **(A)** Schematic of the experiment. Low and high doses of busulfan were administered to young (4–6 weeks) and adult (16–20 weeks) mice on two (LI) and four (HI) consecutive days. Whole BM cells from UBC-GFP transgenic mice were injected into recipients 1 day after busulfan conditioning. Imaging was performed on days 2, 5, and 42 post-HCT; (figure was created with BioRender.com) **(B, C)** Representative longitudinal images of the calvaria BM in young **(B)** and adult **(C)** mice after transplantation; Blue: blood vessel (Rhodamine-B Dextran), Green: GFP + cells; scale bar: 100 μm; **(D)** Quantification of donor GFP + cells in young and adult peripheral blood on day 42 post-HCT; **(E)** Evaluation of donor cell homing to the calvaria BM 2 days post-HCT in young and adult mice. Green: Control, Red: Low Intensity (LI), Blue: High intensity (HI). n ≥ 3, **p* < 0.05, ***p* < 0.01, ****p* < 0.001.

SUPPLEMENTARY VIDEO S1 Representative *ex vivo* Z-stack video of the femur after the cortical bone was shaved *via* cryostat. Red: blood vessel, Purple: bone surface (Alizarin), White: bone surface (Calcein), Blue: bone, Green: GFP + donor cells; scale bar: 500 µm.

SUPPLEMENTARY VIDEO S2 Representative *in vivo* Z-stack video of the calvarium after Rhodamine Dextran injection. Blue: blood vessel, Green: GFP + donor cells; scale bar: 100 µm.

SUPPLEMENTARY VIDEOS S3–S5 Representative leakage in control (day 2; Video 3) and busulfan conditioned (HI) young mice on days 2 (Video 4) and day 42 (Video 5); White: blood vessel; scale bar: 50 µm.

SUPPLEMENTARY VIDEOS S6–S8 Representative leakage in control (day 2; Video 6) and busulfan conditioned (HI) adult mice on days 2 (Video 7) and day 42 (Video 8); White: blood vessel; scale bar: 50 µm.

## References

[B1] Ashizuka S. Peranteau W. H. Hayashi S. Flake A. W. (2006). Busulfan-conditioned bone marrow transplantation results in high-level allogeneic chimerism in mice made tolerant by *in utero* hematopoietic cell transplantation. Exp. Hematol. 34 (3), 359–368. 10.1016/j.exphem.2005.11.011 16543070 PMC1934419

[B2] Barrett A. J. Battiwalla M. (2010). Relapse after allogeneic stem cell transplantation. Expert Rev. Hematol. 3 (4), 429–441. 10.1586/ehm.10.32 21083034 PMC3426446

[B3] Beerman I. Seita J. Inlay M. A. Weissman I. L. Rossi D. J. (2014). Quiescent hematopoietic stem cells accumulate DNA damage during aging that is repaired upon entry into cell cycle. Cell. Stem Cell. 15 (1), 37–50. 10.1016/j.stem.2014.04.016 24813857 PMC4082747

[B4] Cavazzana-Calvo M. Carlier F. Le Deist F. Morillon E. Taupin P. Gautier D. (2007). Long-term T-cell reconstitution after hematopoietic stem-cell transplantation in primary T-cell–immunodeficient patients is associated with myeloid chimerism and possibly the primary disease phenotype. Blood 109 (10), 4575–4581. 10.1182/blood-2006-07-029090 17272510

[B5] Chen Q. Liu Y. Jeong H. W. Stehling M. Dinh V. V. Zhou B. (2019). Apelin+ endothelial niche cells control hematopoiesis and mediate vascular regeneration after myeloablative injury. Cell. Stem Cell. 25 (6), 768–783. 10.1016/j.stem.2019.10.006 31761723 PMC6900750

[B6] Chhabra S. Ahn K. W. Hu Z. H. Jain S. Assal A. Cerny J. (2018). Myeloablative vs reduced-intensity conditioning allogeneic hematopoietic cell transplantation for chronic myeloid leukemia. Blood Adv. 2 (21), 2922–2936. 10.1182/bloodadvances.2018024844 30396912 PMC6234373

[B7] Chicana B. Abbasizadeh N. Burns C. Taglinao H. Spencer J. A. Manilay J. O. (2022). Deletion of vhl in dmp1-expressing cells causes microenvironmental impairment of B cell lymphopoiesis. Front. Immunol. 13, 780945. 10.3389/fimmu.2022.780945 35250971 PMC8889104

[B8] Chiesa R. Veys P. (2012). Reduced-intensity conditioning for allogeneic stem cell transplant in primary immune deficiencies. Expert Rev. Clin. Immunol. 8 (3), 255–266. 10.1586/eci.12.9 22390490

[B9] Christodoulou C. Spencer J. A. Yeh S. C. A. Turcotte R. Kokkaliaris K. D. Panero R. (2020). Live-animal imaging of native haematopoietic stem and progenitor cells. Nature 578 (7794), 278–283. 10.1038/s41586-020-1971-z 32025033 PMC7021587

[B10] Çiftçiler R. Göker H. Demiroğlu H. Aladağ E. Aksu S. Haznedaroğlu İ. C. (2019). Comparison of myeloablative versus reduced-intensity conditioning regimens for allogeneic hematopoietic stem cell transplantation in acute myeloid leukemia: a cohort study. Turk J. Hematol. 36 (2), 88–96. 10.4274/tjh.galenos.2019.2018.0220 PMC6516104 30717586

[B11] Ciurea S. O. Andersson B. S. (2009). Busulfan in hematopoietic stem cell transplantation. Biol. Blood Marrow Transpl. 15 (5), 523–536. 10.1016/j.bbmt.2008.12.489 PMC4261695 19361744

[B12] Cohen H. J. Pieper C. F. Harris T. Rao K. M. K. Currie M. S. (1997). The association of plasma IL-6 levels with functional disability in community-dwelling elderly. J. Gerontol. A Biol. Sci. Med. Sci. 52A (4), M201–M208. 10.1093/gerona/52a.4.m201 9224431

[B13] Connor K. M. Hsu Y. Aggarwal P. K. Capone S. Colombo A. R. Ramsingh G. (2018). Understanding metabolic changes in aging bone marrow. Exp. Hematol. Oncol. 7 (1), 13. 10.1186/s40164-018-0105-x 29796337 PMC5966925

[B14] Down J. Tarbell N. Thames H. Mauch P. (1991). Syngeneic and allogeneic bone marrow engraftment after total body irradiation: dependence on dose, dose rate, and fractionation. Blood 77 (3), 661–669. 10.1182/blood.v77.3.661.661 1991176

[B15] D Souza A. Lee S. Zhu X. Pasquini M. (2017). Current use and trends in hematopoietic cell transplantation in the United States. Biol. Blood Marrow Transpl. 23 (9), 1417–1421. 10.1016/j.bbmt.2017.05.035 PMC5565685 28606646

[B16] Du W. Cao X. (2018). Cytotoxic pathways in allogeneic hematopoietic cell transplantation. Front. Immunol. 9, 2979. 10.3389/fimmu.2018.02979 30631325 PMC6315278

[B17] Ellis S. L. Grassinger J. Jones A. Borg J. Camenisch T. Haylock D. (2011). The relationship between bone, hemopoietic stem cells, and vasculature. Blood 118 (6), 1516–1524. 10.1182/blood-2010-08-303800 21673348

[B18] Ferrucci L. Corsi A. Lauretani F. Bandinelli S. Bartali B. Taub D. D. (2005). The origins of age-related proinflammatory state. Blood 105 (6), 2294–2299. 10.1182/blood-2004-07-2599 15572589 PMC9828256

[B19] Fletcher R. E. Nunes N. S. Patterson M. T. Vinod N. Khan S. M. Mendu S. K. (2023). Posttransplantation cyclophosphamide expands functional myeloid-derived suppressor cells and indirectly influences Tregs. Blood Adv. 7 (7), 1117–1129. 10.1182/bloodadvances.2022007026 36595377 PMC10070372

[B20] Garcia-Perez L. Van Roon L. Schilham M. W. Lankester A. C. Pike-Overzet K. Staal F. J. T. (2021). Combining mobilizing agents with busulfan to reduce chemotherapy-based conditioning for hematopoietic stem cell transplantation. Cells 10 (5), 1077. 10.3390/cells10051077 33946560 PMC8147230

[B21] Green D. E. Adler B. J. Chan M. E. Rubin C. T. (2012). Devastation of adult stem cell pools by irradiation precedes collapse of trabecular bone quality and quantity. J. Bone Min. Res. 27 (4), 749–759. 10.1002/jbmr.1505 22190044

[B22] Griffin J. M. Healy F. M. Dahal L. N. Floisand Y. Woolley J. F. (2022). Worked to the bone: antibody-based conditioning as the future of transplant biology. J. Hematol. OncolJ Hematol. Oncol. 15 (1), 65. 10.1186/s13045-022-01284-6 35590415 PMC9118867

[B23] Gudiol C. Albasanz-Puig A. Cuervo G. Carratalà J. (2021). Understanding and managing sepsis in patients with cancer in the era of antimicrobial resistance. Front. Med. 8, 636547. 10.3389/fmed.2021.636547 PMC8044357 33869250

[B24] Gyurkocza B. Sandmaier B. M. (2014). Conditioning regimens for hematopoietic cell transplantation: one size does not fit all. Blood 124 (3), 344–353. 10.1182/blood-2014-02-514778 24914142 PMC4102707

[B25] Hasegawa Y. Sawada M. Ozaki N. Inagaki T. Suzumura A. (2000). Increased soluble tumor necrosis factor receptor levels in the serum of elderly people. Gerontology 46 (4), 185–188. 10.1159/000022157 10859456

[B26] Ho Y. H. del Toro R. Rivera-Torres J. Rak J. Korn C. García-García A. (2019). Remodeling of bone marrow hematopoietic stem cell niches promotes myeloid cell expansion during premature or physiological aging. Cell. Stem Cell. 25 (3), 407–418. 10.1016/j.stem.2019.06.007 31303548 PMC6739444

[B27] Hooper A. T. Butler J. M. Nolan D. J. Kranz A. Iida K. Kobayashi M. (2009). Engraftment and reconstitution of hematopoiesis is dependent on VEGFR2-mediated regeneration of sinusoidal endothelial cells. Cell. Stem Cell. 4 (3), 263–274. 10.1016/j.stem.2009.01.006 19265665 PMC3228275

[B28] Ishii, M. (2018). Intravital imaging of dynamic bone and immune systems (New York, NY: Springer New York), 1763. Available at: http://link.springer.com/10.1007/978-1-4939-7762-8.

[B29] Itkin T. Gur-Cohen S. Spencer J. A. Schajnovitz A. Ramasamy S. K. Kusumbe A. P. (2016). Distinct bone marrow blood vessels differentially regulate haematopoiesis. Nature 532 (7599), 323–328. 10.1038/nature17624 27074509 PMC6450701

[B30] Kanate A. S. Majhail N. S. Savani B. N. Bredeson C. Champlin R. E. Crawford S. (2020). Indications for hematopoietic cell transplantation and immune effector cell therapy: guidelines from the American society for transplantation and cellular therapy. Biol. Blood Marrow Transpl. 26 (7), 1247–1256. 10.1016/j.bbmt.2020.03.002 32165328

[B31] Kang M. I. Lee W. Y. Oh K. W. Han J. H. Song K. H. Cha B. Y. (2000). The short-term changes of bone mineral metabolism following bone marrow transplantation. Bone 26 (3), 275–279. 10.1016/s8756-3282(99)00265-3 10710001

[B32] Karvunidis T. Chvojka J. Lysak D. Sykora R. Krouzecky A. Radej J. (2012). Septic shock and chemotherapy-induced cytopenia: effects on microcirculation. Intensive Care Med. 38 (8), 1336–1344. 10.1007/s00134-012-2582-4 22584795

[B33] Kebriaei P. Anasetti C. Zhang M. J. Wang H. L. Aldoss I. de Lima M. (2018). Intravenous busulfan compared with total body irradiation pretransplant conditioning for adults with acute lymphoblastic leukemia. Biol. Blood Marrow Transpl. 24 (4), 726–733. 10.1016/j.bbmt.2017.11.025 PMC5902420 29197676

[B34] Kenyon, M., and Babic, A. (2018). The European blood and marrow transplantation textbook for nurses (Cham: Springer International Publishing). Available at: http://link.springer.com/10.1007/978-3-319-50026-3. 31314221

[B35] Kim T. N. Goodwill P. W. Chen Y. Conolly S. M. Schaffer C. B. Liepmann D. (2012). Line-scanning particle image velocimetry: an optical approach for quantifying a wide range of blood flow speeds in live animals. PLoS ONE 7 (6), e38590. 10.1371/journal.pone.0038590 22761686 PMC3383695

[B36] Kondo H. Searby N. D. Mojarrab R. Phillips J. Alwood J. Yumoto K. (2009). Total-body irradiation of postpubertal mice with ^137^ Cs acutely compromises the microarchitecture of cancellous bone and increases osteoclasts. Radiat. Res. 171 (3), 283–289. 10.1667/RR1463.1 19267555

[B37] Kopp H. G. Avecilla S. T. Hooper A. T. Shmelkov S. V. Ramos C. A. Zhang F. (2005). Tie2 activation contributes to hemangiogenic regeneration after myelosuppression. Blood 106 (2), 505–513. 10.1182/blood-2004-11-4269 15817675 PMC1895182

[B38] Kouam P. N. Rezniczek G. A. Adamietz I. A. Bühler H. (2019). Ionizing radiation increases the endothelial permeability and the transendothelial migration of tumor cells through ADAM10-activation and subsequent degradation of VE-cadherin. BMC Cancer 19 (1), 958. 10.1186/s12885-019-6219-7 31619190 PMC6794838

[B39] Kovtonyuk L. V. Fritsch K. Feng X. Manz M. G. Takizawa H. (2016). Inflamm-aging of hematopoiesis, hematopoietic stem cells, and the bone marrow microenvironment. Front. Immunol. 7, 502. 10.3389/fimmu.2016.00502 27895645 PMC5107568

[B40] Kusumbe A. P. Ramasamy S. K. Adams R. H. (2014). Coupling of angiogenesis and osteogenesis by a specific vessel subtype in bone. Nature 507 (7492), 323–328. 10.1038/nature13145 24646994 PMC4943525

[B41] Kusumbe A. P. Ramasamy S. K. Itkin T. Mäe M. A. Langen U. H. Betsholtz C. (2016). Age-dependent modulation of vascular niches for haematopoietic stem cells. Nature 532 (7599), 380–384. 10.1038/nature17638 27074508 PMC5035541

[B42] Lai C. Y. Yamazaki S. Okabe M. Suzuki S. Maeyama Y. Iimura Y. (2014). Stage-specific roles for Cxcr4 signaling in murine hematopoietic stem/progenitor cells in the process of bone marrow repopulation. Stem Cells 32 (7), 1929–1942. 10.1002/stem.1670 24510783

[B43] Lee G. Y. Jeong S. Y. Lee H. R. Oh I. H. (2019). Age-related differences in the bone marrow stem cell niche generate specialized microenvironments for the distinct regulation of normal hematopoietic and leukemia stem cells. Sci. Rep. 9 (1), 1007. 10.1038/s41598-018-36999-5 30700727 PMC6353913

[B44] Li X. M. Hu Z. Jorgenson M. L. Wingard J. R. Slayton W. B. (2008). Bone marrow sinusoidal endothelial cells undergo nonapoptotic cell death and are replaced by proliferating sinusoidal cells *in situ* to maintain the vascular niche following lethal irradiation. Exp. Hematol. 36 (9), 1143–1156. 10.1016/j.exphem.2008.06.009 18718416

[B45] Lo C. C. Fleming H. E. Wu J. W. Zhao C. X. Miake-Lye S. Fujisaki J. (2009). Live-animal tracking of individual haematopoietic stem/progenitor cells in their niche. Nature 457 (7225), 92–96. 10.1038/nature07434 19052546 PMC2820276

[B46] Lucas D. Scheiermann C. Chow A. Kunisaki Y. Bruns I. Barrick C. (2013). Chemotherapy-induced bone marrow nerve injury impairs hematopoietic regeneration. Nat. Med. 19 (6), 695–703. 10.1038/nm.3155 23644514 PMC3964478

[B47] Maryanovich M. Zahalka A. H. Pierce H. Pinho S. Nakahara F. Asada N. (2018). Adrenergic nerve degeneration in bone marrow drives aging of the hematopoietic stem cell niche. Nat. Med. 24 (6), 782–791. 10.1038/s41591-018-0030-x 29736022 PMC6095812

[B48] Mendelson A. Frenette P. S. (2014). Hematopoietic stem cell niche maintenance during homeostasis and regeneration. Nat. Med. 20 (8), 833–846. 10.1038/nm.3647 25100529 PMC4459580

[B49] Montecino-Rodriguez E. Dorshkind K. (2020). Use of busulfan to condition mice for bone marrow transplantation. Star. Protoc. 1 (3), 100159. 10.1016/j.xpro.2020.100159 33377053 PMC7757355

[B50] Nagler A. Shimoni A. (2019). “Conditioning,” in The EBMT handbook. Editors Carreras E. Dufour C. Mohty M. Kröger N. (Cham: Springer International Publishing), 99–107. Available at: http://link.springer.com/10.1007/978-3-030-02278-5_13.

[B51] Naveiras O. Nardi V. Wenzel P. L. Hauschka P. V. Fahey F. Daley G. Q. (2009). Bone-marrow adipocytes as negative regulators of the haematopoietic microenvironment. Nature 460 (7252), 259–263. 10.1038/nature08099 19516257 PMC2831539

[B52] Ogawa T. Kitagawa M. Hirokawa K. (2000). Age-related changes of human bone marrow: a histometric estimation of proliferative cells, apoptotic cells, T cells, B cells and macrophages. Mech. Ageing Dev. 117 (1–3), 57–68. 10.1016/s0047-6374(00)00137-8 10958923

[B53] Porto M. L. Rodrigues B. P. Menezes T. N. Ceschim S. L. Casarini D. E. Gava A. L. (2015). Reactive oxygen species contribute to dysfunction of bone marrow hematopoietic stem cells in aged C57BL/6 J mice. J. Biomed. Sci. 22 (1), 97. 10.1186/s12929-015-0201-8 26498041 PMC4619579

[B54] Preciado S. Muntión S. Rico A. Pérez-Romasanta L. A. Ramos T. L. Ortega R. (2018). Mesenchymal stromal cell irradiation interferes with the adipogenic/osteogenic differentiation balance and improves their hematopoietic-supporting ability. Biol. Blood Marrow Transpl. 24 (3), 443–451. 10.1016/j.bbmt.2017.11.007 29155314

[B55] Rübe C. E. Fricke A. Widmann T. A. Fürst T. Madry H. Pfreundschuh M. (2011). “Accumulation of DNA damage in hematopoietic stem and progenitor cells during human aging,”PLoS ONE. Editor Freitag M., 6. 10.1371/journal.pone.0017487 PMC3049780 21408175

[B56] Saçma M. Pospiech J. Bogeska R. de Back W. Mallm J. P. Sakk V. (2019). Haematopoietic stem cells in perisinusoidal niches are protected from ageing. Nat. Cell. Biol. 21 (11), 1309–1320. 10.1038/s41556-019-0418-y 31685996

[B57] Sengsayadeth S. Savani B. N. Blaise D. Malard F. Nagler A. Mohty M. (2015). Reduced intensity conditioning allogeneic hematopoietic cell transplantation for adult acute myeloid leukemia in complete remission - a review from the Acute Leukemia Working Party of the EBMT. Haematologica 100 (7), 859–869. 10.3324/haematol.2015.123331 26130513 PMC4486220

[B58] Siclari V. A. Zhu J. Akiyama K. Liu F. Zhang X. Chandra A. (2013). Mesenchymal progenitors residing close to the bone surface are functionally distinct from those in the central bone marrow. Bone 53 (2), 575–586. 10.1016/j.bone.2012.12.013 23274348 PMC3674849

[B59] Singh P. Kacena M. A. Orschell C. M. Pelus L. M. (2020). Aging-related reduced expression of CXCR4 on bone marrow mesenchymal stromal cells contributes to hematopoietic stem and progenitor cell defects. Stem Cell. Rev. Rep. 16 (4), 684–692. 10.1007/s12015-020-09974-9 32418119 PMC7395885

[B60] Sipkins D. A. Wei X. Wu J. W. Runnels J. M. Côté D. Means T. K. (2005). *In vivo* imaging of specialized bone marrow endothelial microdomains for tumour engraftment. Nature 435 (7044), 969–973. 10.1038/nature03703 15959517 PMC2570168

[B61] Slayton W. B. Li X. M. Butler J. Guthrie S. M. Jorgensen M. L. Wingard J. R. (2007). The role of the donor in the repair of the marrow vascular niche following hematopoietic stem cell transplant. Stem Cells 25 (11), 2945–2955. 10.1634/stemcells.2007-0158 17656638

[B62] Socié G. Clift R. A. Blaise D. Devergie A. Ringden O. Martin P. J. (2001). Busulfan plus cyclophosphamide compared with total-body irradiation plus cyclophosphamide before marrow transplantation for myeloid leukemia: long-term follow-up of 4 randomized studies. Blood 98 (13), 3569–3574. 10.1182/blood.v98.13.3569 11739158

[B63] Sudo T. Motomura Y. Okuzaki D. Hasegawa T. Yokota T. Kikuta J. (2021). Group 2 innate lymphoid cells support hematopoietic recovery under stress conditions. J. Exp. Med. 218 (5), e20200817. 10.1084/jem.20200817 33666647 PMC7941180

[B64] Van Os R. Konings A. W. T. Down J. D. (1993). Compromising effect of low dose-rate total body irradiation on allogeneic bone marrow engraftment. Int. J. Radiat. Biol. 64 (6), 761–770. 10.1080/09553009314552011 7903344

[B65] Wu J. W. Jung Y. Yeh S. C. A. Seo Y. Runnels J. M. Burns C. S. (2021). Intravital fluorescence microscopy with negative contrast. PLOS ONE 16 (8), e0255204. 10.1371/journal.pone.0255204 34351959 PMC8341626

[B66] Yanada M. Harada K. Shimomura Y. Arai Y. Konuma T. (2022). Conditioning regimens for allogeneic hematopoietic cell transplantation in acute myeloid leukemia: real-world data from the Japanese registry studies. Front. Oncol. 12, 1050633. 10.3389/fonc.2022.1050633 36505853 PMC9732425

[B67] Yanir A. Schulz A. Lawitschka A. Nierkens S. Eyrich M. (2022). Immune reconstitution after allogeneic haematopoietic cell transplantation: from observational studies to targeted interventions. Front. Pediatr. 9, 786017. 10.3389/fped.2021.786017 35087775 PMC8789272

[B68] Yao Z. Murali B. Ren Q. Luo X. Faget D. V. Cole T. (2020). Therapy-induced senescence drives bone loss. Cancer Res. 80 (5), 1171–1182. 10.1158/0008-5472.CAN-19-2348 31932453 PMC7056549

[B69] Zhao D. Wang C. Zhao Y. Shu B. Jia Y. Liu S. (2017). Cyclophosphamide causes osteoporosis in C57BL/6 male mice: suppressive effects of cyclophosphamide on osteoblastogenesis and osteoclastogenesis. Oncotarget 8 (58), 98163–98183. 10.18632/oncotarget.21000 29228681 PMC5716721

[B70] Zhou B. O. Yu H. Yue R. Zhao Z. Rios J. J. Naveiras O. (2017). Bone marrow adipocytes promote the regeneration of stem cells and haematopoiesis by secreting SCF. Nat. Cell. Biol. 19 (8), 891–903. 10.1038/ncb3570 28714970 PMC5536858

[B71] Zou Q. Hong W. Zhou Y. Ding Q. Wang J. Jin W. (2016). Bone marrow stem cell dysfunction in radiation-induced abscopal bone loss. J. Orthop. Surg. 11 (1), 3. 10.1186/s13018-015-0339-9 PMC4704383 26739584

