# Supplementary figures and images for: Age and dose dependent changes to the bone and bone marrow microenvironment after cytotoxic conditioning with busulfan
Source: Front Cell Dev Biol. 2024 Jul 30;12:1441381. doi: 10.3389/fcell.2024.1441381 (PMC11319712; doi:10.3389/fcell.2024.1441381)

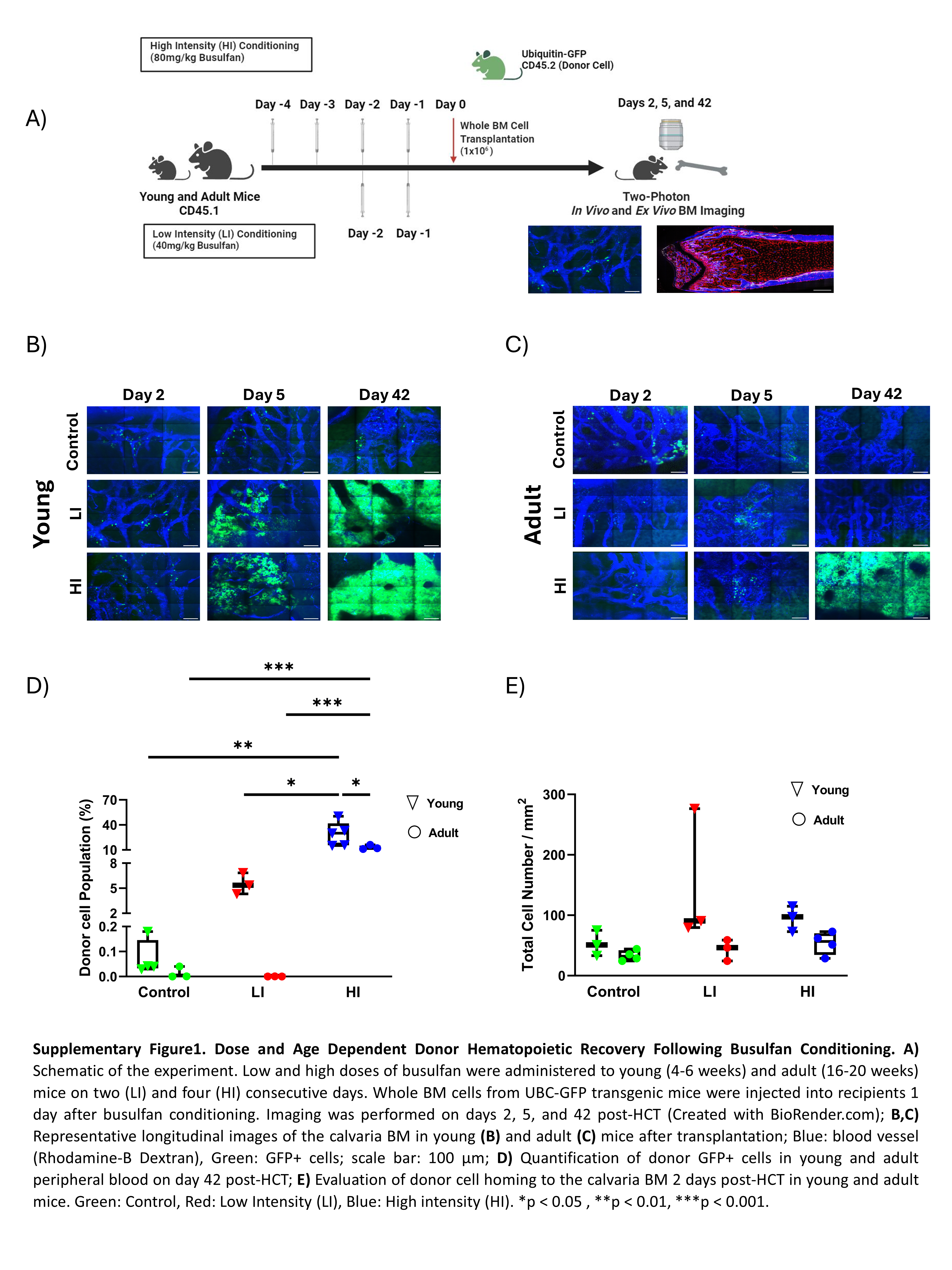

Supplement: Supplementary file 3 [file Image1.tif]
